# Cation Homeostasis in Red Cells From Patients With Sickle Cell Disease Heterologous for HbS and HbC (HbSC Genotype)

**DOI:** 10.1016/j.ebiom.2015.09.026

**Published:** 2015-09-18

**Authors:** A. Hannemann, D.C. Rees, S. Tewari, J.S. Gibson

**Affiliations:** a Department of Veterinary Medicine, University of Cambridge, Madingley Road, Cambridge CB3 0ES, UK; b Department of Paediatric Haematology, King's College London School of Medicine, King's College Hospital NHS Foundation Trust, Denmark Hill, London SE5 9RS, UK

**Keywords:** Sickle cell disease, HbSC, Red cells, Potassium permeability, KCl cotransport

## Abstract

Sickle cell disease (SCD) in patients of HbSC genotype is considered similar, albeit milder, to that in homozygous HbSS individuals — but with little justification. In SCD, elevated red cell cation permeability is critical as increased solute loss causes dehydration and encourages sickling. Recently, we showed that the KCl cotransporter (KCC) activity in red cells from HbSC patients correlated significantly with disease severity, but that in HbSS patients did not. Two transporters involved in red cell dehydration, the conductive channels P_sickle_ and the Gardos channel, behaved similarly in red cells from the two genotypes, but were significantly less active in HbSC patients. By contrast, KCC activity was quantitatively greater in HbSC red cells. Results suggest that KCC is likely to have greater involvement in red cell dehydration in HbSC patients, which could explain its association with disease severity in this genotype. This work supports the hypothesis that SCD in HbSC patients is a distinct disease entity to that in HbSS patients. Results suggest the possibility of designing specific treatments of particular benefit to HbSC patients and a rationale for the development of prognostic markers, to inform early treatment of children likely to develop more severe complications of the disease.

## Highlights

• Red cells from HbSC sickle cell patients showed significant differences in K^+^ permeability cf. those from HbSS patients.
• Conductive potassium pathways, P_sickle_ and the Gardos channel were less active in red cells from HbSC patients.
• KCl cotransport (KCC) had higher activity in HbSC red cells, altered regulation, and correlates with disease severity.
• The genetic, molecular and pharmacological regulation of KCC in HbSC patients is thus of particular clinical relevance.

Sickle cell disease (SCD) is the commonest severe inherited disorder affecting millions of people. Two-thirds of patients are homozygous (HbSS genotype); one-third are heterologous for HbS and HbC (HbSC genotype). Effective treatment remains elusive. Patients' red cells contain the abnormal haemoglobin HbS. Deoxygenated HbS polymerises, initiating clinical sequelae. Sickle red cells lose solutes more rapidly than normal which greatly encourages HbS polymerisation. Red cell permeability is critical. HbSC red cells have significant permeability differences which correlate with disease severity. Understanding regulation of red cell permeability in these patients is thus of particular clinical relevance and may lead to novel treatments.

## 1 Introduction

Sickle cell disease (SCD) is one of the commonest severe inherited disorders affecting millions of people worldwide (Piel et al., 2013). Complications of the disease arise from the presence in patients' red cells of the abnormal haemoglobin (Hb), HbS, which has a single amino acid substitution compared to normal adult Hb, HbA. In HbS, valine replaces glutamic acid in the 6th codon of the β chain, with loss of a negative charge (Bunn and Forget, 1986). On deoxygenation, this substitution allows neighbouring molecules of HbS to adhere, forming rigid polymers which distort the shape of the red cell. The complications of SCD all follow from polymerisation of HbS, although in many cases details of the pathogenesis remain unclear.

About two-thirds of SCD patients are homozygous for HbS (HbSS genotype, or disease, sometimes referred to as sickle cell anaemia, SCA) (Rees et al., 2010). Co-inheritance of a second abnormal Hb, HbC, in which lysine replaces glutamic acid at the same position of the β chain, along with HbS produces the heterologous HbSC genotype (HbSC disease) (Nagel and Lawrence, 1991, Nagel and Steinberg, 2001). HbSC individuals account for about one-third of SCD patients (Nagel and Steinberg, 2001) and thereby represent a sizeable patient cohort. The vast majority of laboratory and clinical studies on SCD, however, including those on red cell cation homeostasis, have been carried out on HbSS patients, with HbSC patients being largely and unjustifiably neglected.

Both HbSS and HbSC disease have profound clinical impact, although those homozygous for HbC (HbCC) are largely asymptomatic (Nagel and Steinberg, 2001). Complications are multiple including chronic anaemia, pain and organ dysfunction with signs dependent on the identity of the affected organ - stroke, acute chest syndrome, nephropathy, osteonecrosis, dactylitis, etc. (Rees et al., 2010, Steinberg et al., 2001, Nagel and Platt, 2001). Between individuals, clinical severity is markedly heterogeneous, with the health of some patients being severely compromised, whilst others present with a less severe disease or even a subclinical course. In many cases it is not understood why. Generally, HbSC disease is milder, though it still presents with significant morbidity (Platt et al., 1991, Nagel et al., 2003). For example, life expectancy of HbSC individuals is markedly reduced (Platt et al., 1994), and some complications of SCD, like proliferative retinopathy (Condon and Serjeant, 1970), are over-represented in HbSC patients.

Intracellular cation homeostasis in red cells is maintained mainly by active movement of Na^+^ and K^+^ via the ATP-driven Na^+^/K^+^-pump coupled with a relatively low passive permeability through various transport pathways. Together these set intracellular [K^+^] at about 100 mM and Na^+^ at about 15 mM (Joyce, 1958). A major feature of red cells from SCD patients, however, is their abnormally high cation permeability. This characteristic is important, as it causes red cells to lose intracellular solutes and shrink, thus elevating the intracellular concentration of HbS ([HbS]). As the lag time to polymerisation of deoxygenated HbS is inversely proportional to a very high power of [HbS] (Eaton and Hofrichter, 1987), any shrinkage markedly increases the likelihood of polymerisation as red cells traverse hypoxic regions of the circulation. Considerable effort has been expended on investigating this high cation permeability (Tosteson et al., 1952, Joiner et al., 1993, Gibson and Ellory, 2002, Lew and Bookchin, 2005), and designing potential inhibitors (eg Stocker et al., 2003), but studies are restricted almost exclusively to red cells from HbSS patients.

**Fig. 1:**
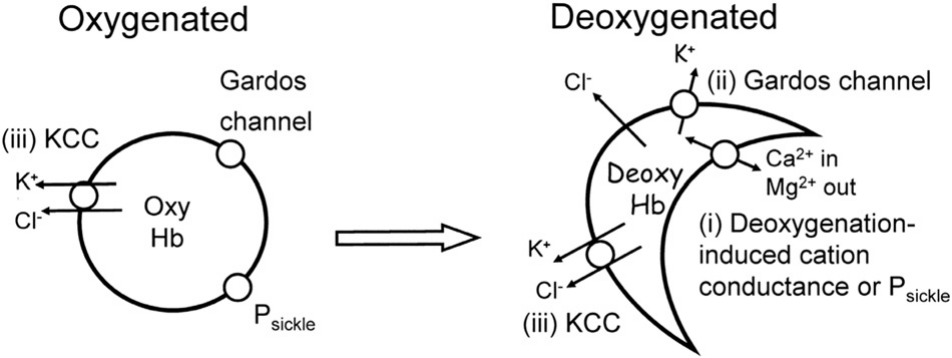
A schematic diagram summarising the main cation pathways involved in solute loss from red cells in patients with sickle cell disease (SCD). In deoxygenated red cells (right), HbS polymerises causing morphological sickling and activation of (i) P_sickle_, a deoxygenation-induced cation conductance. Its main effect is to allow entry of Ca^2 +^ which activates a second transport pathway, (ii) the Ca^2 +^-activated K^+^ channel or Gardos channel, which mediates conductive K^+^ loss at high rates. In oxygenated red cells (left), P_sickle_ and the Gardos channel are inactive. A third transport pathway, (iii) the KCl cotransporter (KCC) mediates obligatorily coupled efflux of K^+^ and Cl^−^. It is present at higher activities in HbS-containing red cells, compared to those from normal (HbAA) individuals. Activity is further enhanced by low pH, swelling and urea. KCC may remain active in deoxygenated sickle cells, with Mg^2 +^ loss via P_sickle_, further increasing its activity. The relative importance of these pathways in red cells from HbSC and HbSS patients appears to differ which may be important in pathogenesis.

Three main transport systems are involved in solute loss and red cell dehydration (summarised in Fig. 1: Lew and Bookchin, 2005): the deoxygenation-induced cation conductance (sometimes termed P_sickle_), the Ca^2 +^-activated K^+^ channel (or Gardos channel) and the KCl cotransporter (KCC). P_sickle_ is activated by deoxygenation and red cell shape change (Tosteson, 1955, Mohandas et al., 1986, Joiner, 1993). It allows entry of Ca^2 +^ (Rhoda et al., 1990) which may then activate the third transporter (Lew et al., 1997), the Gardos channel, with conductive K^+^ loss at high rates, and Cl^−^ following separately through separate anion channels. KCC mediates coupled movements of K^+^ and Cl^−^ (Ellory et al., 1982, Lauf et al., 1992, Gillen et al., 1996). Its activity is abnormally elevated in red cells from HbSS patients (Brugnara et al., 1986, Crable et al., 2005), and it also responds differently to modulatory stimuli such as O_2_ tension (Gibson et al., 1998), when compared to red cells from normal HbAA individuals. It may also be further stimulated by Mg^2 +^ depletion via P_sickle_ (Ortiz et al., 1990, Delpire and Lauf, 1991). As noted above, with the exception of a few studies involving a handful of HbSC patients (Canessa et al., 1986), our understanding of these systems comes from work on red cells from SCA patients. The behaviour of red cells from HbSC patients and management of disease is largely extrapolated from these studies on HbSS - but this may not be justified.

Our recent study comparing clinical parameters and K^+^ transport in red cells from HbSS and HbSC patients indicated significant differences between the two genotypes (Rees et al., 2015). In particular, KCC activity was higher in HbSC patients with more severe forms of SCD (Rees et al., 2015), whilst the same was not true for KCC activity in red cells from HbSS patients. These findings, along with differences in clinical pathology, support the hypothesis that HbSC disease is a distinct clinical entity. Since changes in red cell membrane permeability represent an early event in SCD pathogenesis, with a direct association with HbS polymerisation, further work on membrane transport in red cells from HbSC patients is an imperative. In this report, we characterise more fully the behaviour of the main K^+^ transport systems in red cells from HbSC patients and highlight important differences in comparison with red cells from patients with SCA.

## 2 Materials and Methods

### 2.1 Chemicals

Bumetanide, 3-[*N*-morpholino] propane sulphonic acid (MOPS), nystatin, ouabain, OptiPrep and salts were purchased from Sigma Chemical Co. (Poole, Dorset, UK). Clotrimazole and 4-(2-hydroxyethyl)-1-piperazine ethane sulphonic acid (HEPES) were purchased from Calbiochem (Nottingham, UK). ^86^Rb^+^ was supplied by Perkin Elmer (Beaconsfield, UK).

### 2.2 Sample Collection and Handling

Blood samples were taken for routine tests according to clinical indications from patients at King's College Hospital, homozygous HbSS or heterozygous HbSC for sickle cell disease (SCD), using the anticoagulant EDTA. During the course of this study, around two hundred patients of the HbSC genotype attended the sickle cell clinics at KCH, compared to about four hundred of HbSS genotype. Samples were kept at 4 °C until use within 48 h. The study was approved by the National Research Ethics Committee London-East (reference 11/LO/0065). For some experiments, after routine haematological testing, discarded and anonymised blood was analysed, under the approval of the local ethics committee. All research was conducted in accordance with the Helsinki Declaration of 1975, as revised in 2008.

### 2.3 Solutions and Tonometry

The standard saline (Cl-MBS) comprised (in mM): 145 NaCl, 1.1 CaCl_2_, 5 glucose and 10 MOPS, (pH 7.4 at 37 °C; 290 ± 5 mOsm.kg^− 1^ H_2_O). For experiments in which Cl^−^ dependence of K^+^ influx was examined, NO_3_^−^ containing salts replaced those containing Cl^−^ (N-MBS). The wash solution to remove unincorporated ^86^Rb^+^ comprised isotonic MgCl_2_ (107 mM), buffered with MOPS (10 mM), pH 7.4 at 4 °C (Mg-MBS). Stock solutions of bumetanide (10 mM), ouabain (10 mM) and clotrimazole (CLT; 5 mM) were prepared in 100 mM Tris base, distilled water and DMSO, respectively. In most experiments whole blood was washed five times in N-MBS to remove Cl^−^, plasma and buffy coat. Red cell suspensions at 20% haematocrit (Hct) in N-MBS were placed in tonometers (Eschweiler, Kiel, Germany) flushed with warm, humidified gas mixtures for 20 min at 37 °C to equilibrate at the requisite O_2_ tension before flux measurements and red cell shape analysis (Speake et al., 1997). Gas mixtures were made using a Wösthoff gas mixing pump (Speake et al., 1997). For flux measurements, red cell suspensions were then diluted ten-fold into flux tubes, still equilibrated at the required O_2_ tension. To analyse red cell shape, aliquots of cells were placed in saline containing 0.3% glutaraldehyde before examination under light microscopy.

### 2.4 K^+^ Flux Measurements

To determine the activity of the K^+^ transport pathways, K^+^ influx was measured at 37 °C using ^86^Rb^+^ as a congener for K^+^ (Dunham and Ellory, 1981, Hannemann et al., 2011). Red cells were taken from tonometers and diluted 10-fold into saline, pre-equilibrated at the appropriate O_2_ tension, and, unless otherwise stated, at 260 mOsm.kg^− 1^ and pH 7. ^86^Rb^+^ was added in 150 mM KNO_3_ to give a final [K^+^] of 7.5 mM. Typically, three flux conditions were used: (i) Cl-MBS, (ii) Cl-MBS with clotrimazole and (iii) N-MBS with clotrimazole. Ouabain (0.1 mM) and bumetanide (0.01 mM) were present in all experiments to obviate any K^+^ transport through the Na^+^/K^+^ pump and the Na^+^-K^+^-2Cl^−^ cotransporter, respectively. After incubation with radioisotope for 10 min, red cells were washed five times in ice-cold Mg-MBS wash solution to remove extracellular ^86^Rb^+^. Following the final wash, the cell pellet was lysed with Triton X-100 (0.1%) and protein precipitated with trichloroacetic acid (5%). Activity was then measured as Čerenkov radiation by liquid scintillation (Packard Tri-carb 2100TR). P_sickle_ was assayed as the deoxygenation-induced, clotrimazole-independent K^+^ influx measured in the absence of Cl^−^ (condition iii); Gardos channel activity as the CLT-sensitive (0.005 mM) K^+^ influx (using conditions i & ii); and KCC activity was assayed as Cl^−^ dependent K^+^ influx (using flux conditions ii & iii). For CLT, dissolved in DMSO, appropriate controls were all treated with the same concentration of solvent (0.1% final). Either microhaematocrit determination or the cyanohaemoglobin method was used to measure the Hct with appropriate samples for this taken before the start of each experiment.

### 2.5 Density Separation

Whole blood was washed three-times in HEPES-buffered saline (HBS, comprising in mM: 140 NaCl, 5 KCl, 0.15 MgCl_2_, 10 HEPES, pH 7.4 at room temperature) to remove plasma and buffy coat. Red cells were separated according to density into light, intermediate and dense fractions by centrifugation on gradients of OptiPrep. Stock solution of OptiPrep (60% *w*/*v* iodixanol) was diluted to 40% *w*/*v* in 3xHBS (HBS containing 30 mM HEPES) before diluting further in HBS to produce the desired densities. Densities used depended on the blood samples and were < 1.095 ± 0.001 and > 1.098 ± 0.001 g.ml^− 1^ for HbSC and < 1.089 ± 0.001 and > 1.093 ± 0.002 g.ml^− 1^ for HbSS to recover the light and dense fraction, respectively. 150 μl of loosely packed red cells were layered over 0.4 ml gradient in 1.5 ml tubes and centrifuged at 700 g at 10 °C for 5 min (Denley BR401 bench-top centrifuge, swing-out rotor). Fractions were isolated, washed in HBS and, where necessary, separated on a different gradient in order to obtain the light, intermediate and dense fraction. Light and dense cell fractions were divided into two, with half kept as controls and half treated subsequently with nystatin.

### 2.6 Nystatin Treatment

Density separated red cells were washed three-times in HK-HBS (comprising in mM: 135 KCl, 10 NaCl, 10 glucose, 10 HEPES, pH 7.4 at RT; 290 ± 5 mOsm.kg^− 1^) before treatment on ice for 45 min with nystatin (0.1 mg.ml^− 1^) at 5% Hct in HK-HBS containing 25 mM sucrose. Nystatin was then removed using seven washes with HK-HBS containing sucrose (25 mM) and bovine serum albumin (1 mg.ml^− 1^) at room temperature. Prior to K^+^ influx measurements, nystatin-treated and untreated red cells were washed four times with ice-cold N-MBS, adjusted to 20% Hct. They were then diluted ten-fold into saline for measurement of K^+^ influx, as described above.

### 2.7 Statistics

Results are presented as means ± S.E.M. of n observations in red cell samples taken from different individuals. Where appropriate, comparisons were made using unpaired (Fig. 3, Fig. 4, Fig. 5, Fig. 7) and paired (Fig. 8) two-tailed Student's t-tests. Correlations were made using the Pearson correlation test. The level of significance used was *p* < 0.05.

## 3 Results

### 3.1 Sickling and Conductive K^+^ Transport in Red Cells From HbSC and HbSS Patients

**Fig. 2:**
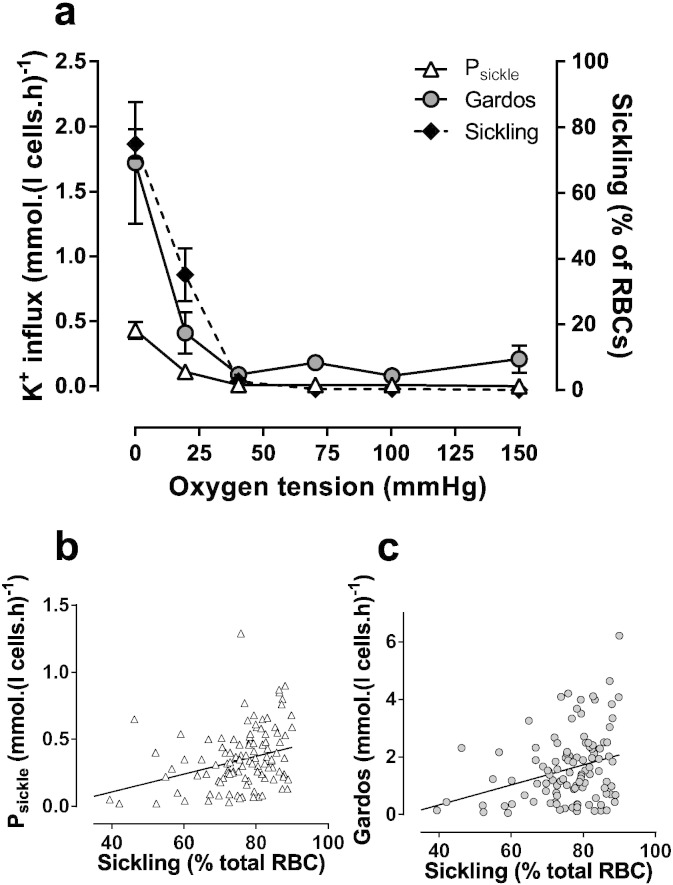
Conductive K^+^ pathways and sickling in red cells from HbSC patients. Red cells (20% haematocrit, Hct) were equilibrated in Eschweiler tonometers at the indicated oxygen tension for 20 min. Red cell aliquots were then fixed in 0.3% glutaraldehyde and percentage sickling assessed by light microscopy. Further aliquots were diluted ten-fold into test tubes for measurement of K^+^ influx (given as mmol K^+^.(l cells.h)^− 1^). P_sickle_ activity is defined as the deoxygenation-induced K^+^ influx in Cl^−^ free saline in the presence of ouabain (100 μM), bumetanide (10 μM) and clotrimazole (CLT; 5 μM), and the Gardos channel activity as the CLT-sensitive K^+^ influx in the presence of ouabain and bumetanide. (a) Oxygen dependence of sickling, P_sickle_ and Gardos channel. Data are presented as means ± S.E.M., n = 4–8. (b, c) Pearson correlation of P_sickle_ and Gardos channel activities with % sickling. Correlations were r = 0.302 (p < 0.01) for P_sickle_ and r = 0.305 (p < 0.01) for the Gardos channel.

Sickling and conductive K^+^ transport were measured in air and across the physiological range of O_2_ tensions in red cells from HbSC patients. Morphological shape change became apparent as O_2_ tension was reduced to about the P_50_ of Hb. Several aspects of K^+^ transport in HbSC cells were also sensitive to O_2_ tension (Fig. 2a). At arterial O_2_ tensions, the deoxygenation-induced cation conductance, or P_sickle_, and the Gardos channel showed low activities. As for sickling, these increased as O_2_ tensions were reduced to levels at which Hb becomes deoxygenated. Activities of both were maximal at the lowest O_2_ tensions and also correlated with degree of cell sickling (Fig. 2b & c: Pearson correlation coefficient r = 0.302, p = 0.0014 for P_sickle_, and r = 0.305, p = 0.0012 for Gardos channel).

**Fig. 3:**
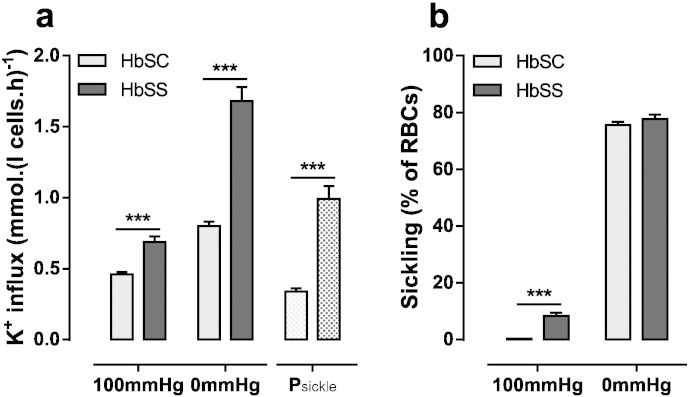
A comparision of P_sickle_ activity and sickling in red cells from HbSS and HbSC patients. Red cells were treated as described in the legend to Fig. 2. (a) K^+^ influx in Cl^−^ free saline in the presence of ouabain (100 μM), bumetanide (10 μM) and CLT (5 μM). P_sickle_ activity is calculated as the difference in K^+^ influx at 0 and 100 mmHg. (b) Sickling (%). Histograms represent means ± S.E.M., n = 110 for HbSC patients and n = 40 for HbSS. *** p < 0.001.

Sickling and the activities of K^+^ transport were compared in red cells from HbSC and HbSS patients in more detail. In oxygenated conditions, the percentage of sickled red cells was 8.4 ± 1.1% and 0.2 ± 0.04% (Fig. 3b) in HbSS and HbSC patients, respectively. The higher values in patients with SCA were presumably due to the presence of irreversibly sickled cells (ISCs) which were observed in oxygenated HbSS samples but were absent in those from HbSC individuals. In both genotypes, sickling was observed in around 80% of red cells on deoxygenation (Fig. 3b).

The residual K^+^ influx in Cl^−^ free media was also measured in both oxygenated and deoxygenated cells in the presence of ouabain and bumetanide (Fig. 3a). At both O_2_ tensions, residual K^+^ influx was lower in red cells from HbSC patients compared to those from HbSS patients. ISCs probably account for the higher levels of residual K^+^ influx in oxygenated red cells from HbSS patients (Fig. 3a). Levels in red cells from both genotypes were increased by deoxygenation, as P_sickle_ becomes activated, more so in the latter than the former (0.99 ± 0.09 mmol.(l cell.h)^− 1^, n = 40, in red cells from HbSS patients cf. 0.34 ± 0.02 mmol.(l cell.h)^− 1^, n = 110, in those from HbSC individuals; p < 0.0001).

**Fig. 4:**
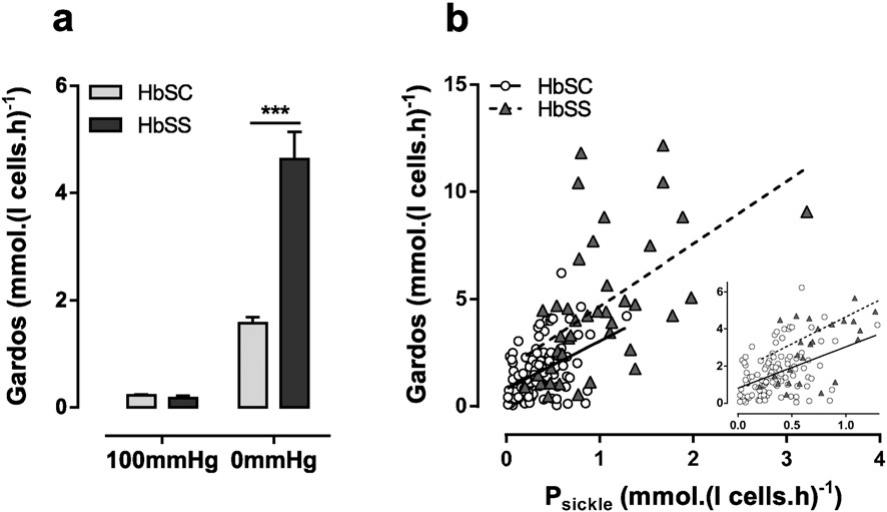
A comparison of Gardos channel activity in red cells from HbSS and HbSC patients. Red cells were treated as described in the legend to Fig. 2. (a) Gardos channel activity (CLT-sensitive K^+^ influx in the presence of 100 μM ouabain and 10 μM bumetanide) in fully oxygenated and fully deoxygenated red cells. Histograms represent means ± S.E.M., n = 110 for HbSC patients and n = 40 for HbSS. ***p < 0.001. (b) Pearson correlation between P_sickle_ and Gardos channel in HbSS and HbSC patients. Insert shows a higher resolution at lower magnitudes of activity of P_sickle_ and Gardos channel. Correlations were calculated as r = 0.433 (p < 0.001) for HbSC red cells and r = 0.530 (p < 0.001) for HbSS.

Gardos channel activity was also measured in fully oxygenated and deoxygenated red cells (Fig. 4a). As for the residual K^+^ influx, it showed higher levels in deoxygenated red cells from HbSS patients compared to those from HbSC individuals (4.63 ± 0.51 mmol.(l cell.h)^− 1^, n = 40, cf. 1.57 ± 0.12 mmol.(l cell.h)^− 1^, n = 110; p < 0.0001). For both genotypes, Gardos channel activity correlated positively with that of P_sickle_ (Fig. 4b: r = 0.433, p < 0.0001 for HbSC; r = 0.530, p = 0.0004, for HbSS), although activities of both were higher in red cells from homozygous HbSS patients compared to those from heterologous HbSC individuals.

**Fig. 5:**
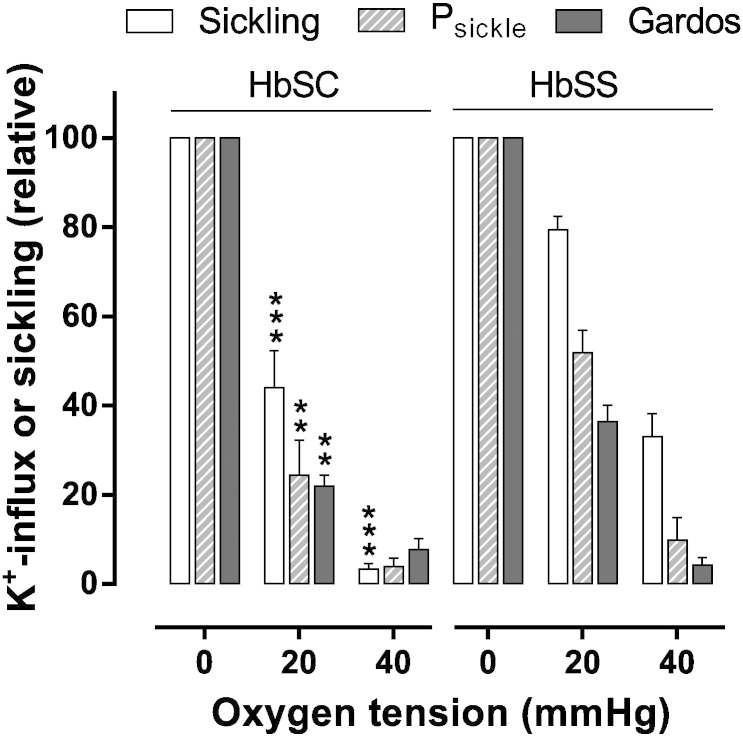
Oxygen dependence of sickling and conductive K^+^ pathways in red cells from HbSS and HbSC patients. Red cells were treated as described in the legend to Fig. 2. Sickling (%), and P_sickle_ and Gardos activities (mmol.(l cells.h)^− 1^), were normalised to values measured at 0 mmHg (which were 75 ± 5%, 0.43 ± 0.06 mmol.(l cells.h)^− 1^ and 1.72 ± 0.47 mmol.(l cells.h)^− 1^, respectively, for red cells from HbSC patients; and 80 ± 2%, 0.82 ± 0.11 mmol.(l cells.h)^− 1^, 3.20 ± 0.47 mmol.(l cells.h)^− 1^ for red cells from HbSS patients. Histograms represent means ± S.E.M., n = 6–8 for HbSC patients and n = 9–12 for HbSS patients. **p < 0.01, *** < 0.001 HbSC cf. HbSS.

At intermediate oxygen tensions, further differences between red cells from HbSC and HbSS patients were apparent (Fig. 5). In both genotypes, full deoxygenation elicits maximal levels of sickling, and activities of P_sickle_ and the Gardos channel. At an oxygen tension of 20 mmHg, just below the P_50_ for Hb, all three parameters were significantly higher in red cells from HbSS patients. A similar trend was also observed at an oxygen tension of 40 mmHg, just above the P_50_ for Hb. These findings suggest that greater levels of hypoxia are required to induce the deleterious morphological changes and K^+^ transport activities in red cells from HbSC patients compared to those from HbSS.

Overall, sickling and activation of conductive K^+^ movement through P_sickle_ and Gardos in red cells from HbSC patients followed the well established pattern observed in red cells from homozygous HbSS patients (Joiner et al., 1993, Lew and Bookchin, 2005, Gibson, 2001). Levels of K^+^ movement through the two channels, however, were significantly reduced in HbSC red cells, consistent with reduced participation of these transport systems in mediating solute loss and red cell dehydration.

### 3.2 Activity and Regulation of the KCl Cotransporter in Red Cells From HbSC and HbSS Patients

Activity of the third transporter, KCC, mediating the obligatorily coupled movement of K^+^ and Cl^−^, was also studied. In red cells from HbSC patients, KCC activity was 0.77 ± 0.54 (n = 3), 1.76 ± 0.26 (n = 6) and 0.44 ± 0.23 mmol.(l cell.h)^− 1^ (n = 6) at saline pHs of 6.5, 7.0 and 7.4, and therefore showed a similar bell-shaped relationship to that seen in red cells from HbSS patients (Brugnara et al., 1986). Hypotonicity (265 mOsm cf. 295 mOsm) also increased KCC activity (1.26 ± 0.4 mmol.(l cell.h)^− 1^ in hypotonic saline cf. 0.44 ± 0.23 in isotonic, n = 6) with the effects of hypotonicty and KCC peak activity at pH 7.0 being additive (2.76 ± 0.38 mmol.(l cell.h)^− 1^, n = 6). The combined stimulus of low pH and swelling was selected for subsequent experiments.

**Fig. 6:**
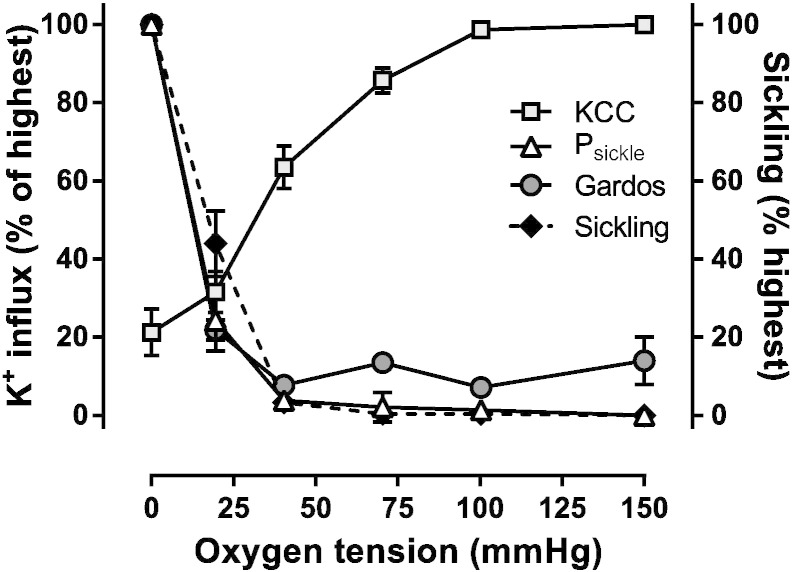
A comparision of the oxygen dependence of KCl cotransport (KCC) activity, with that of sickling, P_sickle_ and Gardos activities, in red cells from HbSC patients. Red cells were treated as described in the legend to Fig. 2. KCC activity was measured as the Cl^−^ dependent K^+^ influx in the presence of ouabain (100 μM), bumetanide (10 μM) and CLT (5 μM), and normalised to that measured at 100 mmHg O_2_ (2.70 ± 0.58 mmol.(l cells.h)^− 1^, n = 4). Percentage sickling and the activities of P_sickle_ and Gardos channel were normalised to values measured at 0 mmHg (75 ± 5% for sickling; 0.43 ± 0.06 mmol.(l cells.h)^− 1^ and 1.72 ± 0.47, n = 7, for P_sickle_ and Gardos channel, respectively). Data are presented as means ± S.E.M..

In the next series of experiments, the O_2_ dependence of KCC activity was investigated. In the case of red cells from HbSC patients, activity of KCC was highest in fully oxygenated red cells. As O_2_ tension was reduced, KCC activity also fell, showing a reciprocal response to that of sickling and activation of P_sickle_ and Gardos channel (Fig. 6 cf. Fig. 2a). In this respect, its behaviour was like that of the transporter in red cells from normal HbAA individuals (Gibson et al., 1998) – although at a considerably higher magnitude overall (Hall and Ellory, 1986). By contrast, in HbSS red cells, although KCC activity initially reduces with O_2_ tension, it reaches minimal activity at about the P_50_ of Hb. It then usually increases again, or remains stable, but does not fall further (Gibson et al., 1998, Hannemann et al., 2014). The O_2_ dependence of KCC in HbSC and HbSS are thus markedly different.

**Fig. 7:**
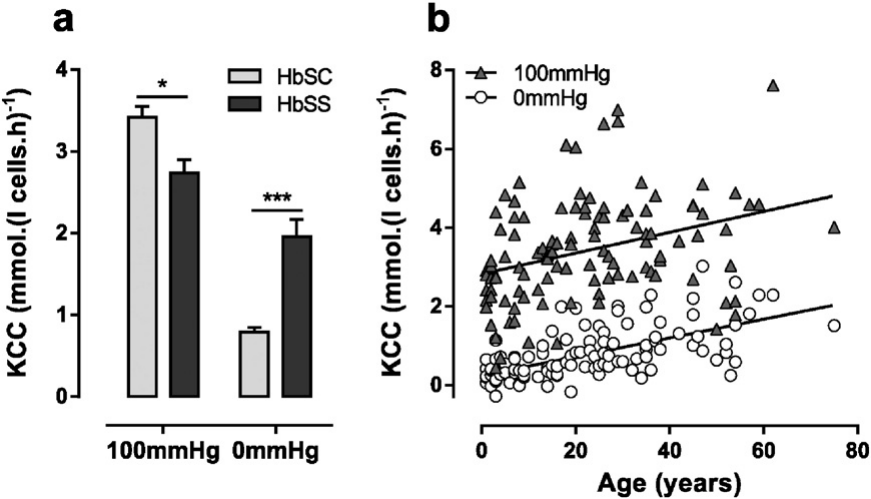
KCC activity in red cells from HbSC and HbSS patients. Red cells were treated as described in the legends to Fig. 2. (a) Cl^−^ dependent K^+^ influx in the presence of ouabain (100 μM), bumetanide (10 μM) and CLT (5 μM). Histograms represent means ± S.E.M., n = 110 for HbSC patients, and n = 40 for HbSS, *p < 0.05, ***p < 0.001. (b) Pearson correlation between KCC activity and age in red cells from HbSC patients. Correlations were calculated as r = 0.339 (p < 0.001) at 100 mmHg O_2_ and r = 0.614 (p < 0.001) at 0 mmHg.

A summary of KCC activity in fully oxygenated (KCC_100_) and deoxygenated (KCC_0_) red cells from HbSC and HbSS patients is presented in Fig. 7a. The difference in O_2_ dependence of KCC activity in red cells from the two genotypes is emphasised, with KCC activity higher in oxygenated red cells from HbSC patients than those from HbSS individuals (3.42 ± 0.79 mmol.(l cell.h)^− 1^, n = 110, in HbSC cf. 2.74 ± 0.16 mmol.(l cell.h)^− 1^, n = 40, in HbSS; p = 0.039), with the opposite pattern being observed under deoxygenated conditions (0.79 ± 0.06 in HbSC cf. 1.96 ± 0.21 in HbSS; p < 0.0001). As well as being significantly higher in oxygenated red cells from HbSC patients, KCC activity was markedly variable between HbSC patients, with a lowest value of 0.44 mmol.(l cell.h)^− 1^ and highest of 7.61. Therefore red cells from some HbSC individuals have particularly high levels of KCC activity, others present with more modest levels. KCC correlated positively with age in both genotypes, especially in KCC_0_ in red cells from HbSC patients (r = 0.614, p < 0.0001). Functionally, KCC mediates volume decrease in reticulocytes during red cell maturation and diminishes with red cell age (Hall and Ellory, 1986) and reticulocyte % correlated to KCC_100_ (r = 540, p < 0.0001) and KCC_0_ (r = 0.28, p < 0.0032) in HbSC patients.

**Fig. 8:**
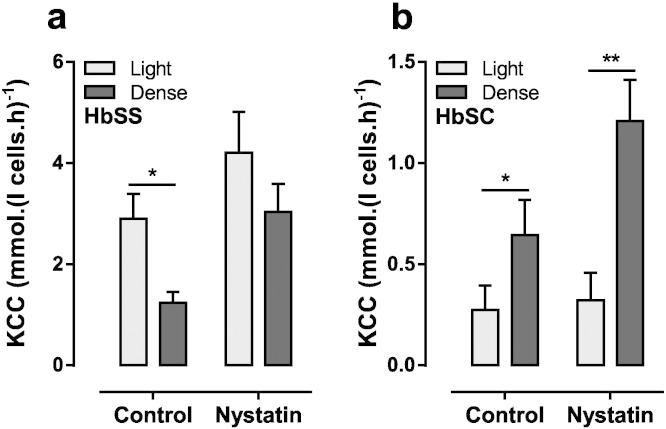
KCC activity in density separated red cell fractions from HbSS and HbSC patients. Red cells were density separated using OptiPrep gradients and either kept on ice or treated with nystatin. They then were suspended at 20% Hct in Cl^−^ free saline before being diluted 10-fold into test tubes for measurement of K^+^ influx. KCC was measured as Cl^−^ dependent K^+^ influx in the presence of ouabain (100 μM), bumetanide (10 μM) and CLT (5 μM). (a) HbSS red cells. (b) HbSC red cells. Histograms represent means ± S.E.M., n = 6. *p < 0.05, **p < 0.01 comparing KCC activity in light and dense fractions.

Increased cell volume is a well known stimulus for KCC activity (Gibson and Ellory, 2003). In normal individuals, younger red cells and reticulocytes are generally larger, less dense and also show higher levels of KCC activity (Hall and Ellory, 1986). Similarly, the least dense red cells from HbSS patients are enriched for reticulocytes and also for higher levels of KCC activity (Brugnara et al., 1986). This occurs despite the existence of so-called “fast-track” dehydrating reticulocytes (Bookchin et al., 1991). The density distribution of red cells in HbSC patients differs. Thus the percentage of reticulocytes in HbSC patients is greater in the denser fractions (Lawrence et al., 1991). In the final set of experiments, therefore, the total red cell populations were separated on a density gradient into dense and light fractions, each containing about a third of the total red cell population, and KCC activity measured in both fractions. In red cells from HbSS patients, KCC activity was higher in the lighter, more swollen fraction of red cells than in the denser one (Fig. 8), as expected. The opposite relationship was observed for red cells from HbSC patients such that, in this case, the denser fraction had greater KCC activity. Red cells from the two density fractions will differ in volume, which will affect the activity of a volume-sensitive transport system like KCC. The two fractions were therefore treated with nystatin prior to measurement of KCC activity, so that both had the same initial volume and intracellular cation content. Following nystatin treatment, the higher levels of KCC activity in the denser fraction of red cells from HbSC patients became more exaggerated. These results are consistent with the postulate that denser circulating red cells in HbSC patients have higher levels of KCC activity.

The above findings are consistent with a more prominent role for KCC in solute loss, dehydration and shrinkage in red cells from HbSC patients – which could explain the correlation between disease severity and KCC activity in this group of patients. By contrast, the conductive K^+^ pathways represented by P_sickle_ and the Gardos channel would appear to be more involved in dehydration of red cells from SCA patients.

## 4 Discussion

The present findings show significant differences in cation homeostasis comparing red cells from HbSC and HbSS patients. The sickling shape change occurred at higher levels of O_2_ tension in red cells from HbSS than HbSC patients, together with activation of the main conductive cation channels, P_sickle_ and the Gardos channel. Both transport pathways showed a similar correlation with sickling. The level of activity of the two channels was significantly lower in HbSC cells compared to HbSS ones, and also required more profound hypoxia to become activated, consistent with a reduced participation of these systems in mediating solute loss and dehydration. By contrast, KCC activity was significantly higher in oxygenated red cells from HbSC patients than those from HbSS individuals. KCC activity varied considerably between HbSC individuals. It also showed a different relationship to O_2_ tension to that observed in red cells from HbSS patients, being inactivated at low O_2_ tension (as seen in normal HbAA red cells). There was a higher level of activity of KCC in denser HbSC red cells compared to that observed in lighter ones. Taken together, these findings are consistent with a greater role for KCC in dehydration of red cells from HbSC patients. They also present a characteristic of red cell membrane transport in HbSC patients which may be important in pathogenesis, and further substantiate the hypothesis that HbSC disease is a different entity to that of homozygous HbSS SCD.

Polymerisation of HbS initiates the clinical complications of SCD (Bunn and Forget, 1986). The resulting sequelae are multiple and diverse, and their individual impact on pathogenesis is difficult to elucidate. Early changes include altered red cell membrane permeability (Gibson and Ellory, 2002, Lew and Bookchin, 2005, Tosteson, 1955, Joiner, 1993). That red cells from SCD patients have elevated cation permeability, which can contribute to disease by mediating solute loss, dehydration and raised HbS concentration, has been established for some time (Tosteson, 1955). The reduced lag time to polymerisation upon deoxygenation observed in shrunken red cells with elevated [HbS] is considered central to disease progression (Eaton and Hofrichter, 1987). Previous reports of cation transport in red cells from HbSC patients have been published but studies were limited to a very small number of individuals (Canessa et al., 1986, Olivieri et al., 1992, Gibson et al., 2001). The present work investigates the behaviour of red cell samples from over a hundred HbSC patients. Of the three transporters involved in dehydration (Lew and Bookchin, 2005), the present findings are consistent with a lesser role for P_sickle_ and Gardos in HbSC disease, whilst supporting a greater involvement of KCC activity (Table 1). The observation that KCC activity in red cells from HbSC patients correlates with frequency of hospitalisation (Rees et al., 2015), a marker of disease severity, emphasises the importance of understanding in detail how this transporter is regulated.

The molecular identity of KCC has been established with four isoforms identified to date, of which three (KCC1, 3 and 4) are found in red cells (Gillen et al., 1996, Pellegrino et al., 1998). In addition, splice variants do occur (Crable et al., 2005), which may be relevant to the different behaviour of KCC in red cells from HbSS and HbSC patients. Physiological regulation of KCC is also complex (Gibson and Ellory, 2003), with evidence for cascades of protein kinases and phosphatases (Cossins et al., 1994), acting on both serine–threonine and tyrosine residues, impacting on transporter activity. This enzymatic regulation is probably key to the differences in response to O_2_ (Gibson et al., 1994, Merciris et al., 2001), perhaps interacting with Hb at the level of the red cell membrane (Sega et al., 2012, Sega et al., 2015). This aspect, however, remains to be fully elucidated.

Maintaining red cell hydration would reduce some of the complications of SCD through reducing the tendency for HbS to polymerise, and represents a longstanding clinical goal (eg Rosa et al., 1980). This is particularly so in red cells from HbSC patients, in which HbS comprises roughly only 50% of the total intracellular Hb. Hydration of these cells would require only a modest increase to reduce the tendency of HbS to polymerise (Fabry et al., 1982). Again, most work has been carried out on red cells from homozygous HbSS patients. To date, the most successful strategy has been identification of reagents which inhibit the Gardos channel. Clotrimazole (Ellory et al., 1992), the *in vitro* inhibitor employed in the current study, cannot be used clinically, as its imidazole ring appears to cause hepatopathy (Brugnara et al., 1996). Analogues such as ICA-17,043 (“senicapoc”) have progressed to clinical trials and were successful at increasing red cell hydration in SCD patients (Stocker et al., 2003, Ataga et al., 2008, Ataga et al., 2011). Their use has been discontinued as they were unable to reduce pain episodes. Partial P_sickle_ inhibitors also exist. They include anion exchange inhibitors such as the stilbenes (Joiner, 1990), but the use of such compounds is precluded by the wide distribution of these transporters through body tissues. Dipyridamole, which is used clinically as an anti-thrombotic compound, also partially reduces P_sickle_ activity (Joiner et al., 2001), and has had some success at reducing clinical signs of SCD (Chaplin et al., 1980, Wun et al., 2013).

No specific inhibitor of KCC has progressed to clinical trials, however, although compounds like H74 were shown to specifically target KCC over the related Na^+^-K^+^-2Cl^−^ cotransporter (NKCC) (Ellory et al., 1990). This molecule, or its related analogues, represent compounds of promise. Simple Mg^2 +^ supplementation has also been used in limited clinical trials, as elevated red cell Mg^2 +^ inhibits KCC activity, with some success (De Franceschi et al., 1997, De Franceschi et al., 2000). If KCC activity is implicated as a key mechanism in pathogenesis, of particular importance in HbSC patients, re-evaluation of potential KCC inhibitors is warranted.

An alternative approach has involved the development of compounds that directly interpolate with HbS molecules, to increase oxygen affinity and to reduce polymerisation upon deoxygenation. Aromatic aldehydes have shown promise and one of them, 5-hydroxymethyl-2-furfural (5HMF), is currently in phase II clinical trials in SCD patients in the US and UK (Abdulmalik et al., 2005, Stern et al., 2012, Health NIH, 2013, Safo and Kato, 2014). We have recently shown that it has additional effects on K^+^ transport, with inhibition of P_sickle_ and Gardos channel and increased hydration, in red cells from SCD patients (Hannemann et al., 2014).

Finally, the marked variability in KCC activity between patients suggests differences of clinical significance in the genetic and molecular control of the transporter when comparing different HbSC individuals. Elucidation of these factors could provide the twin advantages of informing drug design (to inhibit KCC, increase red cell hydration and ameliorate the more severe complications of the disease) and also the possible identification of effective prognostic markers (to inform the management of HbSC patients at an early stage).

## Figures and Tables

**Table 1:** A comparison of sickling and the main potassium permeability pathways in red cells from patients homozygous (HbSS) or heterozygous (HbSC) for sickle cell disease. See text for detailed explanations.

Sickling	Similar in both genotypes
P_sickle_	Reduced in HbSC
Gardos	Reduced in HbSC
KCl cotransport (KCC)	Increased in HbSC
O_2_ dependence of KCC	Increased in HbSC
Density dependence of KCC	Higher in denser HbSC red cells
	Higher in lighter HbSS red cells
